# An Emergent Framework of the Market Food Environment in Low- and Middle-Income Countries

**DOI:** 10.1093/cdn/nzab023

**Published:** 2021-03-13

**Authors:** Djeinam Toure, Anna Herforth, Gretel H Pelto, Lynnette M Neufeld, Mduduzi N N Mbuya

**Affiliations:** Global Alliance for Improved Nutrition, Washington, DC, USA; Helen Keller International, Dakar, Senegal; Harvard TH Chan School of Public Health, Boston, MA, USA; Division of Nutritional Sciences, Cornell University, Ithaca, NY, USA; Global Alliance for Improved Nutrition, Geneva, Switzerland; Global Alliance for Improved Nutrition, Washington, DC, USA

**Keywords:** markets, food environment, measurement framework, low- and middle-income countries, food choice

## Abstract

**Background:**

Food systems are increasingly recognized as critical for advancing nutrition, and the food environment is viewed as the nexus between those systems and dietary consumption. Developing a measurement framework of the market food environment is a research priority, particularly for low- and middle-income countries (LMICs), which face rapid shifts in markets, dietary patterns, and nutrition outcomes.

**Objectives:**

In this study, we sought to assess current conceptions and measures of the market food environment that could be adapted for use in LMICs.

**Methods:**

We conducted a narrative review of the literature to identify measures of the market food environment in recent use. First, we identified and reviewed frameworks of the food environment for LMICs with a specific focus on the market food environment. Second, we compiled 141 unique measures of the market food environment from 20 articles into a list that was pile-sorted by 5 nutrition experts into domains. We then categorized the measures based on percentage agreement across all sorts. Finally, we compared measured and conceptual domains of the market food environment to identify measurement gaps and needed adaptations.

**Results:**

Conceptual frameworks provide differing definitions of the market food environment but conform in their definitions of food availability, price, marketing, and product characteristics. Greater clarity is needed in defining relevant vendor and product characteristics. Eight measured domains of the market food environment emerged from the literature review, with significant overlap among conceptual domains. Measurement gaps exist for food quality, safety, packaging, desirability, and convenience. Personal characteristics also emerged as measured domains, although these are not part of the food environment per se.

**Conclusions:**

These results are a step toward elucidating how, why, and where we measure the market food environment in LMICs. Future research should focus on prioritizing the most meaningful methods and metrics and on developing new measures where gaps exist.

## Teaser

The food environment is the nexus between food systems and dietary consumption. This study elucidates how, why, and where we measure the market food environment in low- and middle-income countries.

## Introduction

Over the last decade, consensus has grown regarding the need to improve food systems for nutrition. After the Scaling Up Nutrition movement introduced the term “nutrition-sensitive development” in 2010, the 2013 *Lancet* series for maternal and child health underlined the need for nutrition-sensitive approaches that align the priorities of food production and supply systems with nutrition (1). In the same year, the Global Panel on Agriculture and Food Systems for Nutrition (henceforth, Global Panel) was created to help governments develop evidence-based policies targeted at various levels of the food system to improve nutrition outcomes. In parallel, the International Congress for Nutrition renewed its commitments to developing food-based dietary guidelines to serve the health, agriculture, and education sectors in shaping healthier food systems (2). The vast majority of the world's poor are net buyers of food (3), and people are purchasing an increasing share of their food globally. It therefore follows that food markets and retail points play a role in determining the quality of diets (4). However, little is known about the market environment in which people make most of their food choices, especially in low- and middle-income country (LMIC) contexts.

Conventional wisdom often assumes effective market forces, i.e., that supply and demand for nutritious foods are balanced and that consumer choice responds to and drives this balance. The reality is considerably more complex. There is a convergence of issues in markets that may affect food choice. On the one hand, the physical and temporal availability of food (5), and the sociodemographic characteristics in a context (6), dictate food accessibility and affordability (7). On the other hand, cultural preference, convenience of preparation or consumption, and desirability, which may also be rooted in aspirational qualities, all further influence how people choose food (8).

The food environment, defined as the interface between the food system and the individual, is a useful concept to understand the complexities of the interactions between food systems and nutrition (9). Food systems encompass the entire range of actors and activities related to the production, aggregation, processing, distribution, consumption, and disposal of food products (10). The food environment is the context in which individuals encounter foods and make decisions on which foods to consume. Multiple types of food environments, such as the home, work, and school food environments, have been characterized in high-income contexts (11–13). This article focuses on the market food environment, the spaces in which consumers make choices on what foods to purchase and consume. This is in view of the rapid change in food procurement which is trending toward purchase of foods in both urban and rural areas of LMICs rather than procurement from people's own production. Currently, data do not exist that enable us to definitively understand how all factors of the market food environment, specifically, influence individual choices and diets in LMICs. The measures needed to unpack these relations are further lacking. Metrics to quantify features of the market food environment will be needed to track changes over time and determine the impact of interventions that aim to improve diet through markets and food retail.

The objective of this work was therefore to identify existing concepts and measures of the market food environment that can be used or adapted for use in LMICs. The specific objectives were 3-fold. First, we sought to identify areas of convergence and divergence in existing conceptual frameworks of the market food environment for LMICs. Second, we sought to identify existing measures of the market food environment published in the literature and the domains that they represent. Finally, we compared the domains present in conceptual frameworks with those measured in the literature in order to identify measurement gaps, provide concrete suggestions for future application in studies of the market food environment, and identify research gaps.

## Methods

Two methods were used to meet the research objectives. First, we conducted a narrative review (14) of the literature to identify *1*) conceptual frameworks of the food environment in LMICs and *2*) existing measures of the market food environment that have been used globally and may be adapted for use in LMICs. The conceptual frameworks we reviewed were similar in intended scope, and all aimed to define the broader food environment in the context of food and nutrition programming for LMICs. We reviewed broad frameworks of the food environment with the objectives of identifying and focusing on the market. Our review of measures was not intended to be exhaustive but instead aimed to capture the main methodological tools and constructs that have been used to describe food environments. Our search of measures was not limited to LMICs to ensure that we could capture a breadth of methodological approaches and given that few studies of the food environment have been conducted overall. We focus our discussion on how measures may be used or adapted for LMIC contexts, specifically. For an exhaustive list of measures of the food environment see Lytle and Sokol, 2017 (15).

Second, we extracted unique measures of the market food environment reported or presented in the literature and conducted a pile-sort with key informants to identify measured domains of the market food environment. Pile-sorting is a methodology in which respondents are asked to group similar items into piles, which allows the respondents, rather than the researchers, to determine similarity and salience (16). The aim of the pile-sort was to identify measured domains, or categories, of the food environment that are salient across the different epistemologies of nutrition.

### Search strategy and literature review

Peer-reviewed articles and agency reports identified through online searches were the primary sources of literature and were complemented with bibliography reviews. First, we conducted a PubMed search of the medical subject headings (MeSH) terms “food environment” AND “nutrition” AND “diet.” A total of 428 studies were returned and 158 were retained after title screening. Animal studies and articles that did not refer to the food environment or any aspect of it (e.g., retail locations) were excluded. In a second screening, studies were excluded if they did not present a measure of the food environment; were focused solely on school, work, or home food environments; or assessed food environment policy measures (17, 18). A total of 109 studies were retained.

Second, we identified additional articles using a snowball approach, which entailed reviewing the bibliographies of the initial set of articles to identify relevant peer-reviewed articles and agency-developed tools. Finally, given the variable language used to describe and measure the food environment or aspects of it (e.g., store characteristics, food venue location), a series of additional searches were conducted on PubMed by using a combination of the following (non-MeSH) terms: “consumer OR community OR nutrition OR store OR food OR food venue AND environment OR nutrition environment”. An additional 35 articles were identified in this manner.

### Pile-sorting and data extraction

Measures of the food environment were extracted from 20 articles. Only unique measures were extracted to present the breadth of measures available. Where studies used the same or very similar measurements, the measure was only recorded once. For each study, we compiled a list of all individual measures used to assess the food environment. Where indexes were described, only the individual questions or observations constituting the index were listed. In total, 141 measures were compiled. The list was shared with 5 nutrition experts, purposively approached because of their documented expertise in the areas of food environment, ethnographic methods, drivers of food choice, dietary assessment, and value chains research. The experts represented a breadth of epistemological positioning within the field of nutrition, which ensured that the sorting of measures considered different perspectives across the field of nutrition. The list of items was shared with the experts in random order on an online platform: Proven By Users. The experts were asked to group items into an unspecified number of piles so that similar items were in the same pile. A follow-up discussion was conducted with each expert to understand the reasoning behind his or her sorts. Two experts (AH and GHP) who participated in the pile-sort are coauthors of this article. The methods deployed in this study primarily involved a narrative review of the literature, coupled with an analysis of data extracted from published articles. As such, they did not meet the definition of research with human subjects and consequently, were not submitted for ethical review.

### Data analysis

Two of the authors (DT and MNNM) reviewed the piles and identified emergent domains based on the percentage agreement between the assignment by experts. Reviewers were blind to the individual sorting decisions of the 5 experts and analyzed the aggregate percentage agreements among items. All piles with ≥80% item agreement were reviewed and discussed by the authors to determine the emergent construct of the food environment captured by the measures. Any individual items with <80% agreement (*n *= 11 items) to a given pile were reviewed individually and categorized into an existing pile. Within piles, subcategories were identified where items reached 100% agreement. Once all domains and subdomains were identified and considering errors made during sorting that experts mentioned in the pile-sorting exit interviews, individual items that did not logically belong to a domain were reclassified (*n *= 3 items).

## Results

### A review of the food environment frameworks for LMICs

We identified 6 conceptual frameworks of the food environment specific to LMICs and reviewed 5 (2, 9, 19–21). The framework by Vandevijvere and Swinburn (18) was excluded because it focused solely on policies that affect the food environment.

Herforth and Ahmed (19) define the market food environment according to 4 operational domains: the availability, affordability, desirability, and convenience of foods. They define availability as the quantity and presence of foods in markets and affordability as the price of these foods relative to consumer incomes. Desirability in this article refers to food quality, advertising, status of foods, and other external, not individual, valuations of food. Convenience is defined as the time associated with acquiring, preparing, and consuming foods. These authors later provided an updated definition (21), further defining desirability as “promotion and quality,” to disambiguate it from differing use of the same term by other scholars (5), and adding sustainability properties as an additional operational domain. They describe a typology of food environments: wild, cultivated, informal markets, and formal markets.

The Global Panel (22) provides a conceptual framework of the food environment nested in a socioecological model (23) and describes the food environment as a set of food characteristics (e.g., quality, taste, physical access to food, prices, promotion, and labeling). The environment is influenced by food supply chains and influences consumer behavior but remains distinct from both, in line with Herforth and Ahmed's conceptualization.

The High-Level Panel of Experts (HLPE) framework defines the food environment as the physical, economic, political, and sociocultural context in which consumers engage with the food system to acquire, prepare, and consume food. This framework emphasizes physical proximity, affordability, food promotion and advertising, quality, and safety as domains of the food environment that dictate food choice. The domain of proximity accounts for both an individual's mobility to access food and the physical position of food resources relative to his or her home.

Turner et al. (9) build on several of these frameworks and describe the food environment as having 2 interrelated categories: the external and the personal food environment. Domains of the external food environment include food prices, availability, product and vendor characteristics, and marketing and regulation in line with the HLPE framework. A set of parallel domains comprise the personal food environment: affordability, accessibility, desirability, and convenience. Desirability in this article refers to individuals’ personal preferences and excludes the inherent characteristics of the food, in contrast to previous work (19). Aspects of food quality, nutrient content, and safety are instead conceptually distinguished from desirability and constitute a separate domain: product characteristics. Turner et al. describe convenience as the relative effort to prepare, cook, and consume a product, a definition that provides a distinction from the time needed to acquire foods (accessibility).

### Synthesis and comparison of framework domains

**Table 1** demonstrates the overlap and definitional differences in the domains included across the 5 conceptual frameworks. The overlap reinforces the centrality of 4 conceptual domains of the food environment: food availability, food price and affordability, promotion (e.g., location in store, advertisement, labeling), and product characteristics (e.g., nutrient content, safety, packaging). Safety, convenience, processing, and packaging are inconsistently defined and highlighted by the various frameworks. In addition, vendor characteristics are mentioned only by the Turner et al. framework. Some domains are defined differently in various frameworks (**Supplemental Table 1**).

**TABLE 1:** Overlap and definitional differences of domains in 4 conceptual frameworks of the food environment in low- and middle-income countries^1^

Domain	Turner et al. (9)	Herforth and Ahmed (19)	Downs et al. (21)	Global Panel (22)	High-Level Panel of Experts (20)	Consistent definition across frameworks
Accessibility	X			X	X	X
Affordability	X	X	X	X	X	X
Desirability	X	X				
Convenience	X	X	X			
Availability	X	X	X	X		X
Price	X			X		X
Product characteristics	X	X		X		
Quality	X		X	X	X	
Safety	X				X	
Taste	X			X		
Packaging	X					
Processing	X					
Sustainability properties			X			
Promotion/marketing	X		X	X	X	

1 Domains marked as present in the framework must have been explicitly named and described as central to the food environment. References to domain names as examples or in the broader discussion of the article were not included.

Individual consumer characteristics (e.g., taste, transport, knowledge) that influence people's individual interactions with the food environment are discussed in the presentation of all frameworks, pointing to their importance in considering how food environments affect food choice and diet. However, these characteristics are largely distinct from the environment itself in the frameworks by the Global Panel (2) and Herforth and Ahmed. The personal characteristics that determine people's experience of the food environment—and even the forces that shape the food environment (e.g., food supply chain)—are of public health significance but the food environment remains distinct from both.

Only Downs et al. (21) specify the context in which food is acquired in LMICs. They discuss 2 overarching types of food environments: natural and built. Natural food environments include wild (such as forests and oceans) and cultivated food environments (such as gardens and orchards); built food environments include informal markets (such as street vendors) and formal markets (such as restaurants and supermarkets). The authors further discuss patterns of how food environments change, aligned with the nutrition transition. Creating typologies of food sources was also done in high-income country contexts (24), but these were not all applicable for LMIC contexts.

The following section reviews the available metrics of the market food environment. We did not review metrics of wild or cultivated food environments, which are discussed in Downs et al., and which are relatively scarce.

### Emergent domains of the measured market food environment

Few studies have attempted to characterize market food environments in LMICs as a whole in an effort to facilitate understanding of consumers’ food purchases and choice (25). Studies have instead measured individual aspects of market food environments. As a result of this, most studies from which measures were extracted were from high-income countries. **Table 2** presents the full set of articles and measures.

**FIGURE 1:**
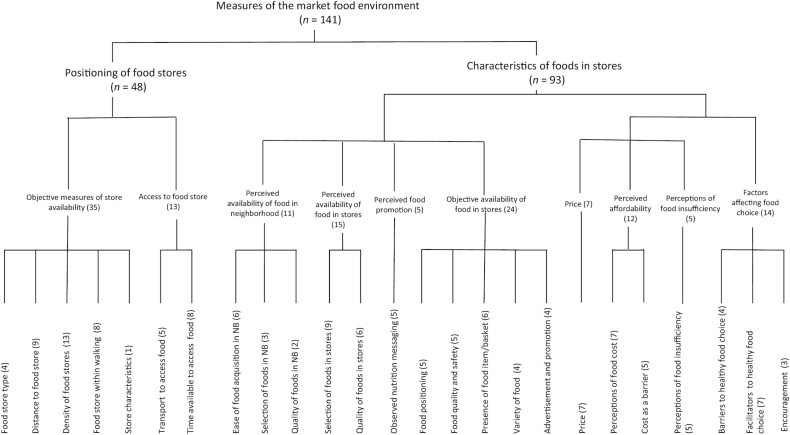
Clustered dendrogram of measures of the market food environment from the literature review, categorized into domains. This dendrogram is based on the sorting of 141 measures of the market food environment by 5 experts in the field of nutrition. Ten domains emerged from the pile-sort exercise based on piles of items with ≥80% agreement. These include objective availability of food retail locations (*n *= 35 items), access to food retail (*n *= 13 items), perceived availability of food near home (*n *= 11 items), perceived availability of food at a retail location (*n *= 15 items), perceived nutrition messaging (*n *= 5 items), objective availability of food at a retail location (*n *= 24 items), price (*n *= 7 items), affordability (*n *= 12 items), perceptions of food insufficiency (*n *= 5 items), and factors affecting food choice (*n *= 14 items). Each domain contains further subcategories. The domains are separated into 2 overarching categories: 1 related to the position of food retail in space and a second related to the characteristics of foods encountered inside a market or retail location. NB, neighborhood.

Ten domains, based on piles of items with 80% agreement, emerged from the pile-sort exercise (**Figure 1**). These included objective availability of food retail locations (*n *= 35 items), access to food retail (*n *= 13 items), perceived availability of food near home (*n *= 11 items), perceived availability of food at a retail location (*n *= 15 items), perceived food promotion (*n *= 5 items), objective availability of food at a retail location (*n *= 24 items), price (*n *= 7 items), affordability (*n *= 12 items), perceptions of food insufficiency (*n *= 5 items), and factors affecting food choice (*n *= 14 items). The domains separated into 2 overarching categories: 1 related to the position of food retail in space and a second related to the characteristics of foods encountered inside a market or retail location.

#### Positioning of food retail locations

The 2 domains addressing availability of food retail and access to food retail clustered closely together under the overarching category pertaining to the positioning of food retail (Figure 1). First, the domain addressing availability of food retail primarily included objective measures of food store density, food store or vendor types in a specific area, distance of food stores to participants’ homes, and the presence of stores within walking distance. Second, the domain addressing access to food retail contained subcategories of physical and temporal access to stores. Physical access was composed of measures related to car ownership, presence of public transportation, or the ability to shop within the neighborhood (26, 27). Temporal access included measures of personal travel time, childcare availability during shopping, and proximity to a food store—a primary driver of store choice.

#### Characteristics of food at retail

Eight domains with 80% item agreement emerged relative to the characteristics of foods at retail: perceived availability of food near home, perceived availability of food at a retail location, perceived food promotion, objective availability of foods at a retail location, food price, affordability, perceptions of food insufficiency, and factors affecting food choice (Figure 1).

Domains addressing perceived availability of food near home and at retail, nutrition messaging, and objective measures of food in stores clustered closely together. Measures of perceived food availability at retail and near home (all questions assessed on a Likert scale) included subcategories addressing the selection and quality of available food. Quality measures referred to produce freshness, and selection to the availability of different food groups or specific items. The domain addressing perceived food promotion included 4 measures, which assessed the extent to which consumers notice nutrition labeling and promotion when purchasing foods (28).

**TABLE 2:** Compendium of measures of the food environment extracted from the literature^1^

Authors	Measure	Response categories	Data collection method
Moore et al. (29)	Lack of access to adequate food shopping is a problem in my neighborhood	4-point Likert	Consumer survey
	A large selection of fruits and vegetables is available in my neighborhood	4-point Likert	Consumer survey
	A large selection of low-fat products is available in my neighborhood	4-point Likert	Consumer survey
Rose and Richards (30)	Density of supermarkets within a given distance from a participant home	Number	Spatial mapping
	Distance to store where most of food is purchased	Km	Consumer survey
	Ownership of a car	Yes/no	Consumer survey
	Travel time to store where most of food is purchased	Min	Consumer survey
	Where do you buy most of your food?	Supermarket type	Consumer survey
Gustafson et al. (31)	A large selection of fruits and vegetables is available in my primary food store	5-point Likert	Consumer survey
	A large selection of low-fat meat products is available (90% lean beef, skinless chicken) in my primary food store	5-point Likert	Consumer survey
	A large selection of brown breads is available in my primary food store	5-point Likert	Consumer survey
	A large selection of low-fat cheese or skim milk is available in my primary food store	5-point Likert	Consumer survey
	A large selection of low-fat products is available in my neighborhood	Min	Consumer survey
	The fruits and vegetables in my neighborhood are high quality	Miles	Consumer survey
	Travel time to primary food store from participant home	Yes/no for each SIC type	Consumer survey
	Distance to primary food store from participant home	Count	Spatial mapping
	Number of food stores by type within an area (census tract)	Count	Spatial mapping
	Number of food stores by type within participant activity space (measured by GPS tracking of consumer movement)	Count	Spatial mapping
Gustafson et al. (32)	Number of healthy food stores within participant activity area	Count	Spatial mapping
	Number of unhealthy food stores within participant activity area	Count	Spatial mapping
	Little variety in types of foods that can be purchased	5-point Likert	Consumer survey
	Food prices are high	5-point Likert	Consumer survey
Sharkey et al. (33)	How would you rate the variety of fruits and vegetables at this store?	5-point Likert	Consumer survey
	How would you rate the freshness of fruits and vegetables at this store?	5-point Likert	Consumer survey
	How would you rate the price of fruits and vegetables at this store?	4-point Likert	Consumer survey
	The food we bought last month didn't last and we didn't have enough money to buy more	4-point Likert	Consumer survey
	We couldn't afford to buy balanced meals	Yes/no	Consumer survey
	Did any adult have to cut the size of their meal because there wasn't enough?	Number, yes/no count	Consumer survey
	Distance between participant home and nearest supermarket	Miles	Store audit
	Distance between participant home and nearest good source of fresh fruits and vegetables	Miles	Spatial mapping
	Distance between participant home and nearest good source of fresh or processed fruits and vegetables	Miles	Spatial mapping
	Availability of fresh fruits and vegetables in store	Yes/no by food FFV type	Consumer survey
	Variety of fresh fruits and vegetables in store	Count	Consumer survey
Zenk et al. (34)	Selection of fresh fruit and vegetables at the store where they shopped	4-point Likert	Consumer survey
	Quality of fresh fruit and vegetables at the store where they shopped	4-point Likert	Consumer survey
	Affordability of fresh fruit and vegetables at the store where they shopped	4-point Likert	Consumer survey
Morland et al. (35)	Location of primary food store	Urban/suburban	Spatial mapping
Williams et al. (36)	I do not buy many fruit because they cost too much	5-point Likert	Consumer survey
	I do not buy many vegetables because they cost too much	5-point Likert	Consumer survey
	During the past year, how often did members of your family (including spouse/partner) eat healthy low-fat foods with you?	5-point Likert	Consumer survey
	During the past year, how often did members of your family (including spouse/partner) encourage you to eat healthy low-fat foods?	5-point Likert	Consumer survey
	During the past year, how often did members of your family (including spouse/partner) discourage you from eating unhealthy foods?	5-point Likert	Consumer survey
Lucan et al. (27)	Do you HAVE to travel outside of your neighborhood to go to a supermarket?	Yes/no	Consumer survey
	How would you rate the overall quality of groceries available in the stores in your neighborhood?	5-point Likert	Consumer survey
	How easy or difficult is it for you to find fruits and vegetables in your neighborhood?	4-point Likert	Consumer survey
	Availability of conventional or limited-assortment supermarket within a census tract	Yes/no	Spatial mapping
	Availability of conventional or limited-assortment supermarket within a quarter mile beyond	Yes/no	Spatial mapping
	Presence of a subway trolley in participant neighborhood	Yes/no	Consumer survey
Inglis et al. (26)	How much do you consider the cost of food when deciding what food or groceries to buy when food shopping?	4-point Likert	Consumer survey
	How much do you consider “specials/discounts/sales” when deciding what food or groceries to buy when food shopping?	4-point Likert	Consumer survey
	I do not buy many fruits because they cost too much	4-point Likert	Consumer survey
	Have you ever run out of food in the last 12 mo and been unable to afford more?	Yes/no	Consumer survey
	I do not buy many vegetables because they cost too much	5-point Likert	Consumer survey
	I can do most of my food shopping at stores in my local neighborhood	5-point Likert	Consumer survey
	At the shop where I buy fruits and vegetables, the variety of fresh fruits and vegetables is limited	5-point Likert	Consumer survey
	The fresh produce in my area is usually of a high quality	5-point Likert	Consumer survey
	There are lots of healthy options for eating out in my local neighborhood	5-point Likert	Consumer survey
	Difficulties with transport to and from my usual place of food shopping	Yes/no	Consumer survey
	Access to a motor vehicle for private use	Yes/no	Consumer survey
	Access to childcare if they needed to go shopping without children	Yes/no	Consumer survey
	Presence of a supermarket within walking distance	Yes/no	Consumer survey
	Fruit and vegetable store within walking distance	Yes/no	Consumer survey
	Small grocery store within walking distance	Yes/no	Consumer survey
	Fresh-food mart within walking distance	Yes/no	Consumer survey
	Fast-food restaurant within walking distance	Yes/no	Consumer survey
	Non-fast-food restaurant within walking distance	Yes/no	Consumer survey
	Café within walking distance	Yes/no	Consumer survey
Dubowitz et al. (37)	Approximately how much money do you spend per week on food?	USD	Consumer survey
	How many people does the amount of money you spend on food feed?	Number of people	Consumer survey
	Distance from home to the nearest food store	Miles	Spatial mapping
	Distance from home to the nearest full-service supermarket	Miles	Spatial mapping
	Which food type dominates the shelf view?	Fruits; vegetables; sugar- sweetened beverages; candy or sweetened baked goods; salty snacks; or other	Store audit
Caldwell et al. (38)	How easy or difficult is it for you to get fresh produce (fruits and vegetables)?	4-point Likert	Consumer survey
	Depth and width of linear space devoted to fruits and vegetables	Inches × inches	Store audit
	Proportion of produce that is fresh	All fresh, nearly all fresh, most fresh, some fresh, most not fresh	Store audit
	Produce basket price (lowest price for apples, potatoes, orange juice, apple sauce, green beans, and frozen corn)	USD	Store audit
	Minimum price for any fresh produce (of 16 oz)	USD	Store audit
Glanz et al. (39)	Price per pound for the cheapest item in 10 indicator food categories [fruit, vegetables, milk, ground beef, hot dogs, frozen dinners, baked goods, beverages (soda/juice), whole-grain bread, and baked chips]	USD price for fruit, vegetables, milk, ground beef, hot dogs, frozen dinners, baked goods, beverages (soda/juice), whole-grain bread, and baked chips	Store audit
	Price per pound for the “healthier” vs. “regular” item in 10 indicator food categories [fruit, vegetables, milk, ground beef, hot dogs, frozen dinners, baked goods, beverages (soda/juice), whole grain bread, and baked chips]	USD price for fruit, vegetables, milk, ground beef, hot dogs, frozen dinners, baked goods, beverages (soda/juice), whole-grain bread, and baked chips	Store audit
	Availability of any food item in 10 indicator food categories [fruit, vegetables, milk, ground beef, hot dogs, frozen dinners, baked goods, beverages (soda/juice), whole-grain bread, and baked chips]	Available/unavailable	Store audit
	Number of varieties of foods within 10 indicator food categories [fruit, vegetables, milk, ground beef, hot dogs, frozen dinners, baked goods, beverages (soda/juice), whole-grain bread, and baked chips]	Number of varieties within a food group	Store audit
	Observation of the majority of a given type of fruits or vegetables as being clearly bruised, old looking, overripe, or spotted?	Acceptable/unacceptable condition of food group	Store audit
Block and Kouba (40)	Availability of 102 foods commonly eaten by low-income households and meeting Federal dietary guidelines and Food Guide Pyramid serving sizes (fresh fruits and vegetables, frozen FV, canned FV, breads and grains, dairy, fats and oils, baby food and formula, sugars and sweets, spices and baking supplies)	Available/unavailable	Store audit
	Price of the least expensive food item at a selected size (fresh fruits and vegetables, frozen FV, canned FV, breads and grains, dairy, fats and oils, baby food and formula, sugars and sweets, spices and baking supplies)	USD	Store audit
Cheadle et al. (41)	The proportion of poultry and fish in the meat display	Proportion of linear width	Store audit
	The proportion of reduced-fat milk in the milk display	Proportion of linear width	Store audit
	The proportion of 100% whole-wheat bread in the bread display	Proportion of linear width	Store audit
	Presence or absence of health promotion items (for produce, milk, eggs, and bread)	Yes/no	Store audit
Green and Glanz (28)	Thinking about the store where you buy most of your food, how do you usually travel to this store?	Car or other	Consumer survey
	About how long would it take to get from your home to the store where you buy most of your food, if you walked there?	4-point Likert	Consumer survey
	How important are each of the following factors in your decision to shop at the store where you buy most of your food: near or on the way to the other places I spend time	4-point Likert	Consumer survey
	How important are each of the following factors in your decision to shop at the store where you buy most of your food: near home	4-point Likert	Consumer survey
	It is easy to buy fresh fruits and vegetables in my neighborhood	5-point Likert	Consumer survey
	It is easy to buy low-fat products, such as low-fat milk or lean meats, in my neighborhood	5-point Likert	Consumer survey
	The low-fat products in my neighborhood are of high quality	5-point Likert	Consumer survey
	There is a large selection of low-fat products available in my neighborhood	5-point Likert	Consumer survey
	How important are each of the following factors in your decision to shop at the store where you buy most: selection of foods	4-point Likert	Consumer survey
	How important are each of the following factors in your decision to shop at the store where you buy most: quality of foods	4-point Likert	Consumer survey
	How important are each of the following factors in your decision to shop at the store where you buy most: prices of foods	4-point Likert	Consumer survey
	At the store where you buy most of your food, how would you rate the price of fresh fruits and vegetables?	4-point Likert	Consumer survey
	I often buy food items that are located near the register	5-point Likert	Consumer survey
	The unhealthy foods are usually located near the end of the aisles	5-point Likert	Consumer survey
	I often buy items that are at eye level on the shelves	5-point Likert	Consumer survey
	There are lots of signs and displays encouraging me to buy the unhealthy foods	5-point Likert	Consumer survey
	I notice signs that encourage me to purchase healthy foods	5-point Likert	Consumer survey
	I see nutrition labels or nutrition information for most packaged food at the stores	5-point Likert	Consumer survey
Burgoine and Monsivais (11)	Density of food outlets within 1-km street network buffer of home	Count by food store type (cafés/coffee shops, convenience stores, entertainment, health and leisure, restaurants, specialist stores, supermarkets, takeaways)	Spatial mapping
	Density of food outlets within 1-mile street network buffer of home		
	Density of food outlets within 1-km street network buffer of work		
	Density of food outlets within 1-mile street network buffer of work		
	The shortest street network distance for commuting journeys on foot and by bike/car between home and store	Km	Spatial mapping
	The shortest street network distance for commuting journeys on foot and by bike/car between work and store	Km	Spatial mapping
Timperio et al. (42)	Presence/absence of food outlet by type (cafés/restaurants; fast food; supermarkets/grocery stores; convenience store; greengrocer; and butcher, seafood, or poultry retailer)	Count	Spatial mapping
de Menezes et al. (43)	Times store open for business	Times of day	Spatial mapping
	Days store open for business	Days of week	Spatial mapping
	Number of food stores by type: *1*) large-chain supermarkets; *2*) specialized FV markets or open-air food markets; *3*) local grocery stores; and *4*) convenience stores and bakeries, within 1600-m buffer	Number	Spatial mapping
	Availability of ≥1 item for the 5 most frequently purchased ultra-processed foods (including sugar-sweetened beverages, chocolate cookies, and processed corn snacks)	Count	Store audit
	Availability of ≥1 item for the 20 most frequently consumed fruits and vegetables	Count	Store audit
	Number of varieties of each item available (20 most frequently consumed fruits and vegetables and 5 most commonly purchased ultra-processed foods)	Count	Store audit
	Presence or absence of signs or advertisements that encouraged to purchase products (for FV and ultra-processed foods)	Present/absent	Store audit
	The average price of 20 FV and 5 ultra-processed foods	USD	Store audit
	Hygienic and sanitary aspects of the food stores (the presence of trash, animals, dust, and stagnant water; the condition of the floor, walls, doors, windows, and ceiling; and the adequacy of lighting, ventilation, and waste management)	Present/absent	Store audit
	Self-efficacy to change FFV consumption across 4 situations: affordability	5-point Likert	Consumer survey
	Self-efficacy to change FFV consumption across 4 situations: availability	5-point Likert	Consumer survey
	Self-efficacy to change FFV consumption across 4 situations: time to prepare	5-point Likert	Consumer survey
	Self-efficacy to change FFV consumption across 4 situations: consume recommended amount	5-point Likert	Consumer survey
	Taste as a reason for FV intake	5-point Likert	Consumer survey
	Availability of time to buy FV as a reason for FV intake	5-point Likert	Consumer survey
	Ease of preparation as a reason for FV intake	5-point Likert	Consumer survey
	Health as a reason for FV intake	5-point Likert	Consumer survey
	High expense as a reason against FV intake	5-point Likert	Consumer survey
	Dislike FV as a reason against FV intake	5-point Likert	Consumer survey
	Not having time to eat as a reason against FV intake	5-point Likert	Consumer survey
	No social support as a reason against FV intake	5-point Likert	Consumer survey
Pessoa et al. (44)	Presence of food store (mini-market/grocery stores/warehouses; supermarkets and hypermarkets; shops and produce open-air markets; mobile food shops; restaurants; bars; eateries)	Count by type	Spatial mapping
Duran et al. (45)	Presence of the 10 most frequently purchased fruits and vegetables, and the 3 most frequently consumed ultra-processed foods: sugar-sweetened beverages, chocolate sandwich cookies, and corn chips	Available/unavailable	Store audit
	Number of varieties available in food group (10 most frequently purchased fruits and vegetables, and the 3 most frequently consumed ultra-processed foods: sugar-sweetened beverages, chocolate sandwich cookies, and corn chips)	Count	Store audit
	Observation of >75% of the available produce as bruised, old looking, overripe, or spotted	Acceptable/unacceptable condition of food group	Store audit
	Price of cheapest item in the 10 most frequently purchased fruits and vegetables, and the 3 most frequently consumed ultra-processed foods: sugar-sweetened beverages, chocolate sandwich cookies, and corn chips	USD	Store audit
	The number of different signs or advertisements that promoted the purchase of fruits and vegetables or ultra-processed foods	Count	Store audit

1 FFV, fresh fruits and vegetables; FV, fruits and vegetables; GPS, Global Positioning System; SIC, Standard Industrial Classification.

The domain addressing objective availability of food at retail included subcategories pertaining to food placement; quality and safety; the physical presence of food items or food basket items; variety; and advertisement. Food placement (*n *= 5 items) included measures of shelf space allocated to a specific food or the proportion of a food display dedicated to an item. Food quality referred to the observed freshness of produce and to aspects of safety and hygiene (*n *= 1 item). Freshness measures were based on visual assessments of the quality of fruits and vegetables as bruised, rotten, or acceptable (33, 34). The physical presence of food was measured by checking the availability of food items against predetermined lists of common items, known as market baskets, and via taking a census of all available foods. For instance, Donkin et al. (46) devised a list of 71 foods that are common and acceptable to a local ethnically diverse population to audit food availability in low-income communities of London. In Brazil, Menezes et al. (47) measured the availability of the 5 most purchased ultra-processed foods, and the 20 most common fresh fruits and vegetables. This approach is an adaptation of the Nutrition Environment Monitoring Survey developed in the United States, which measures the availability of common “healthy” and “unhealthy” foods in retail environments. Other market baskets have been developed based on national dietary guidelines (48) or thrifty-eating plans (40). Measures of food advertisement and promotion (*n *= 424 items) included the absence or presence of promotional materials for food items or food groups.

Domains pertaining to food price, affordability, perceptions of food insufficiency, and other factors affecting food choice clustered closely together. Price was measured as an absolute figure, per unit, and was also reported comparatively, for healthier compared with less healthy options (47) and for the most common and cheapest food items in a food category (39). Price indexes have also been formulated to reflect the cost of an adequately diverse diet and adequately nutritious diet and the lowest-cost nutritious diet based on available foods.

Items in the domain of affordability measured on a Likert scale consumer perceptions of food cost (*n *= 7 items) and perceptions of cost as a barrier to consumption of foods within a specific group (*n *= 5 items). Items related to food security, meal size and frequency, and total food expenditure figured in the domain addressing perceptions of food insufficiency. Factors affecting food choice included measures of encouragement for specific food intake as well as barriers to and facilitators of healthy food intake (e.g., self-efficacy, taste).

### Synthesis of domains represented by existing measures of the market food environment

Of the 10 measured domains presented here that reached 80% item agreement in pile-sorting, we deem only the following to be measures of the market food environment: availability of food retail, food availability at retail (both perceived and objective measures), food price (perceived and objective), and food promotion. These measured domains of the food environment include both objective (i.e., availability of a food retail store, food availability at retail, price) and subjective subdomains and measures (i.e., perceived availability of food near home or at retail, perceived food promotion, perceived cost).

Food environment research often includes measures of consumer characteristics that are relevant to but outside of the food environment, notably individual access to food stores, factors affecting food choice, and perceptions of food insufficiency.

### Review of the conceptual domains and available measures of the food environment

There is significant agreement in the domains represented in frameworks of the food environment and the domains measured in food environment research. However, gaps remain in the completeness of the available measures of product characteristics (including quality, safety, and packaging), retail or vendor types, food marketing, desirability, and convenience. Furthermore, adaptations of existing measures are needed to determine their relevance to LMIC markets. Below, we compare the conceptual domains of the food environment with the available measures and identify gaps.

#### Availability

Measures of food availability dominated the list of food environment measures in the literature. These included both objective and subjective measures of food at retail and the availability of food retail locations. The use of measures such as checklists and shelf space to determine the presence and quantity of food items at retail has applications in LMICs, as do measures of food retail density and location. However, studies adapting these measures are needed. Adaptations of measures of food availability in LMICs using audit or market basket checklists will need to consider the dietary patterns of the specific population and the contribution of different foods to local diets.

The availability of digital information on food store location and consumer home or other activity space has allowed for the wide breadth of studies investigating food store density and its relation to diet in high-income countries (11, 15, 49, 31). In LMICs, digital information on food retail location is not widely available, and the definition of a market space in informal settings poses an additional challenge to research. Unlike in high-income countries, where individual stores are clearly identifiable formal structures in which people purchase a large share of their food, there is not a strict operational definition of a market in LMICs, given the informal nature of food vending. Defining relevant market or activity spaces within which the density and types of food retail available to consumers can be meaningfully assessed will be an important next step in further market research for LMICs.

#### Food price

We found multiple measures of food prices in markets, which is consistent with the prominence of this domain among conceptual frameworks in the literature on the food environment. These included both objective and subjective measures of food cost.

Price indexes that have been calculated for LMICs tend to rely on secondary data, often generated from government sentinel market monitoring (e.g., West African Agricultural Market Information System Network and Vulnerability Assessment Mapping). These data can be useful for understanding food prices at national level, and depending on the country may be high-resolution enough to characterize regional and district levels (50). However, they can be difficult to access from governments, and sometimes exclude many nutritious food items (in favor of staples). Describing individual markets or small local food environments may require primary data collection, which can have significant cost and labor implications. In informal markets common in many LMICs, price is not consistently displayed and units of sale are not always standardized. Collecting price data may therefore require interviewing vendors and standardizing units of sale, a process that is costly and labor intensive. Perceived cost may also be an effective way of measuring prices in some settings or for some research questions.

#### Product characteristics

Despite differences in definitions, all frameworks highlighted the importance of certain product characteristics and, to a lesser extent, vendor characteristics. Product characteristics included aspects of food quality, safety, processing, and packaging.

Measures to describe vendor characteristics identified in the literature included assessments of store type and opening hours (47). Measures for other potentially important characteristics of informal markets, such as market days and provision of credit, did not appear in the studies. Analyses with vendor type data have identified areas of low food access (37) and of low-quality food store access (51). Similar characterizations in LMICs would provide an initial indication of the types of retail environments that different populations have access to.

The measures of product characteristics identified in the literature pertained to food quality and food safety. Although all frameworks included food quality, safety, and packaging as part of the food environment, measures for these constructs were sparse. We identified 1 observation-based measure of hygiene and related infrastructure. This included assessments of the presence of trash, animals, dust, and stagnant water and the condition of the floor, walls, doors, and windows in markets (43). Measures of food packaging and processing were absent altogether. Only 1 question about the convenience of available foods was found. Additional measures are needed to characterize quality beyond freshness, as are additional measures of perceived and objective food safety.

#### Food promotion

Nine measures relating to marketing and advertisement were identified. These included objective measures of product arrangement and of the presence of certain types of labeling and signs. Research has also measured consumers’ perceptions of promotional materials and their influence on purchase, accounting for an additional 5 measures. The extent to which consumers notice nutrition labeling or purchase foods that are at eye level or are prominently displayed has also been measured (28). Data in the literature on food marketing and advertising practices focus mainly on countries where the nutrition transition (the shift in dietary choices that occurs with demographic and socioeconomic change) is well underway and where overconsumption of energy-dense foods contributes to poor nutrition outcomes.

Adaptations for LMICs will vary by context and the predominant dietary issues that a population faces. As noted above, in urban areas and where high-energy, low-nutrient foods are increasingly displacing nutritious foods or leading to overconsumption, understanding the marketing forces that affect consumer food choice serves public health interests. Similarly, in urbanizing areas where diets are transitioning, monitoring these types of changes is important. Market observation tools that assess the predominance of shelf space and consumer-oriented questions that assess the influence of marketing on food choice can be easily adapted in survey modules.

### Domains outside of market food environments

Our review identified individual characteristics that are not part of the food environment per se, but are important in understanding how consumers interact with the food environment. These include perceptions of food insufficiency, and access to food retail, which includes personal mobility and availability of time to access food stores.

We identified measures of an individual's physical and temporal access to food—easily adaptable survey questions that can be administered in LMICs. Affordability was largely measured via perception-based questions on the cost of food and cost as a barrier to obtaining food (28, 40, 52). Only 1 measure of desirability, a score based on the sensory aspects of food items, emerged in our review. However, broader measures of a food's desirability or the factors that render a food desirable within a cultural context are lacking.

## Discussion

In this article, we reviewed frameworks and measures of the market food environment and highlighted the utility of the concept to understand and monitor how food systems “deliver nutrition” through markets. Through our review, we identified areas of consensus among the frameworks in terms of the key domains of the market food environment: availability, price and affordability, promotion, and product characteristics. We presented and organized previously uncollated measures of the market food environment that can be adapted for future use in LMICs. Finally, we compared the conceptual and measured domains of the food environment to reveal gaps in the available measures of each domain and in adaptations of existing measures needed for use in LMICs. This work is an initial step toward the development of a measurement framework and tools that can be used to characterize market food environments in LMICs.

It is important to acknowledge that the food environment is inherently intangible and therefore difficult to define. The food environment is a concept defined relative to the ways that people interact with the food system and reflects cultural norms and preferences, economic conditions, and geography, all of which change over time. Although “food environment” is inconsistently defined in the literature, the concept of a food environment is valuable as an organizing framework that facilitates research on critical features and determinants of food intake and nutrition. Furthermore, it guides exploration of the cognitive frameworks (e.g., perception of desirability) that dictate food choice and that can be modified to promote more positive dietary outcomes.

Building on the aforementioned concepts, we defined the market component of the food environment as the context in which consumers interact with and purchase food created by a consortium of complex social, economic, and physical factors. This definition is in line with the socioecological model of the food environment that places environments between individual factors and larger systems (19). From a measurement perspective, we highlighted the availability of foods at retail, food promotion price, and quality of foods, as well as the availability of food retail and vendors, as key domains of the market food environment. The 8 key domains where metrics were developed, encouragingly, included both objective and perceived measures. Assessing the food environment through both objective and perceived measures could yield greater and different insights from either alone. This operationalization of the market food environment is a consensus between explicit frameworks of the broader food environment for LMICs and the frameworks implicit in studies across different country contexts that have measured aspects of the market food environment.

Adaptations to the measures presented in this review, largely from high-income countries, are required to determine the relevance of those measures to LMICs. Where gaps were identified, new measures must be developed. We noted the need for greater specificity of relevant product characteristics and for better measures of these characteristics. In particular, measures of food quality, safety, and packaging are lacking, as are measures of vendor typologies. Furthermore, the cost of collecting primary data in the types of informal markets that are common in many LMICs must be considered.

The market food environment affects consumer diet through interaction with individual characteristics—notably purchasing power and food preferences and tastes—as well as physical and temporal access to markets. Although these factors are not part of the market food environment per se, they are also fundamental determinants of how the food environment affects consumer food choice and diet. We also noted very few measures of desirability.

We have identified 5 priority areas for future research:

1. *Develop audit or market-basket tools* that capture a variety of foods or food groups needed for a healthy diet, as well as those detrimental to health given the current dietary trends in LMICs.
2. *Develop vendor typologies relevant to LMICs*. Developing store or vendor typologies for LMICs is a low-cost adaptation of measures presented in this article that can help characterize the types of foods that consumers have access to in a geographical location. The measures identified here can be developed into a market observation survey module that captures information on vendor and food characteristics that offer a more complete characterization of informal markets, which dominate food retail in LMICs but for which data to date are lacking.
3. *Develop measures of food quality, safety, and packaging as well as measures of convenience and desirability* relevant to LMICs. All of these factors play a role in shaping food choice. The nutrition transition, characterized by a shift from traditional diets to foods high in meat, sugar, and fats and rising rates of overweight and obesity, is underway in many LMICs. Aspects of packaging, convenience of food preparation, and desirability have been successfully leveraged by the private sector to increase the consumption of low-nutrient-density, high-calorie, obesogenic foods as studies of the nutrition transition in other contexts have shown. Understanding aspects of convenience, packaging, and desirability is key to prevent the overconsumption of these foods and to promote the consumption of nourishing foods. Further, food-borne illness is a significant source of disease and disability. Transitions toward food retail in LMICs are outpacing food safety infrastructure and regulation, and better knowledge of food safety characteristics and valuations are needed.
4. *Research the association between perception-based measures and objective measures of the market food environment*. This will help to elucidate where the highest-impact interventions might be, either in knowledge, attitudes, and practices; or in objectively improving the food environment; or both.
5. *Conduct assessments of informal markets that collect information on all domains of the market food environment, especially in contexts of urban and rural poverty in LMICs*. The vast majority of studies reviewed were from high-income countries and there is very little complete information on the market food environments of LMICs. Most studies reviewed here investigated individual domains of the food environment and as such cannot speak to the relative effects or importance of other domains on consumer food choice. Future studies should seek to characterize the environment as a whole and explore the utility of a composite food environment index in addition to individual domain measures. Above all, better assessments of informal market environments are needed to guide efforts to improve the acquisition and consumption of nutritious foods.

Little is known about the impact of interventions targeting transport, processing, and retail on market food environments, even without considering their effects on consumption. The private-sector actors involved in the production, processing, transport, and retail of foods represent potentially important points of intervention to improve food access and nutrition. Operationalizing the framework of the market food environment is a first step in building the evidence base to determine the private sector's current impact on diet and the potential for private-sector interventions to improve diets and nutrition.

In conclusion, the concept of the food environment provides an organizational framework to examine the impact markets have on consumer food choice and diet. There is conceptual consensus among frameworks that availability, price, food characteristics, and promotion are key domains of the market food environment. There are gaps in available measures of food and vendor characteristics, notably food quality, safety, packaging, convenience, and desirability. Further research is needed to adapt promising measures to LMICs and to develop new measures where they do not exist.

## Supplementary Material

nzab023_Supplemental_File

## Notes

Supported by a Government of the Netherlands grant (to DT).

Author disclosures: the authors report no conflicts of interest.

Supplemental Table 1 is available from the “Supplementary data” link in the online posting of the article and from the same link in the online table of contents at https://academic.oup.com/cdn/.

Abbreviations used: HLPE, High-Level Panel of Experts; LMIC, low- and middle-income country; MeSH, medical subject headings.
