# Supplementary material for: An Emergent Framework of the Market Food Environment in Low- and Middle-Income Countries
Source: Curr Dev Nutr. 2021 Mar 13;5(4):nzab023. doi: 10.1093/cdn/nzab023 (PMC8075774; doi:10.1093/cdn/nzab023)
Supplement: nzab023_Supplemental_File [file nzab023_supplemental_file.docx]

**ANNEX**

**Table A1: Domains and definitions presented in three food environment frameworks for low- and middle-income countries**

| **Domain** | **Definition** |
| --- | --- |
| **Turner et al. (9)** | |
| Accessibility | Physical distance, space and place, mode of transport, activity space |
| Affordability of food | Purchasing power |
| Desirability | Preferences, acceptability, taste |
| Convenience | Relative time and effort to prepare and consume foods |
| Availability of foods | Presence of a food vendor or product |
| Price | Monetary value |
| Marketing and regulation | Promotional info, labelling, advertising, policies |
| Product and vendor characteristics | Vendor properties, food quality, safety, packaging, shelf life |
| **Herforth and Ahmed (18)*** | |
| Affordability of food | Price, purchasing power |
| Convenience | Time to prepare and quire foods |
| Desirability | External factors (status of foods, cultural norms, advertising, product placement, food quality). Excludes taste and personal preferences |
| Availability of food | Physical presence of food |
| **Global Panel (21)**** | |
| Nutrient quality of available food | |
| Taste of available food | |
| Physical access to food | |
| Price | |
| Food promotion | |
| Labelling | |

***Herforth and Ahmed referred to the following domains as relevant but outside the food environment: taste and internal elements of desirability. ** The Global Panel referred to the following domains as relevant but outside the food environment: purchasing power, agricultural production subsystems, food storage and transport, food transformation systems, food retail subsystems.
